# Neuromuscular Factors Contributing to Reductions in Muscle Force After Repeated, High-Intensity Muscular Efforts

**DOI:** 10.3389/fphys.2019.00783

**Published:** 2019-06-24

**Authors:** Benjamin J. C. Kirk, Gabriel S. Trajano, Timothy S. Pulverenti, Grant Rowe, Anthony J. Blazevich

**Affiliations:** ^1^Centre for Exercise and Sports Science Research, School of Medical and Health Sciences, Edith Cowan University, Joondalup, WA, Australia; ^2^School of Exercise and Nutrition Sciences, Faculty of Health and Institute of Health and Biomedical Innovation, Queensland University of Technology, Brisbane, QLD, Australia; ^3^Department of Physical Therapy, College of Staten Island, Staten Island, NY, United States

**Keywords:** neuromuscular fatigue, excitation–contraction coupling, corticospinal excitability, persistent inward currents, caffeine

## Abstract

Multiple neuromuscular processes contribute to the loss of force production following repeated, high-intensity muscular efforts; however, the relative contribution of each process is unclear. In Experiment 1, 16 resistance trained men performed six sets of unilateral isometric plantar flexor contractions of the right leg (3 s contraction/2 s rest; 85% maximal voluntary contraction torque; 90-s inter-set rest) until failure with and without caffeine ingestion (3 mg kg^-1^) on two separate days. Corticospinal excitability and cortical silent period (cSP) were assessed before and immediately, 10 and 20 min after the exercise. In Experiment 2, electrically evoked tetanic force and persistent inward current (PIC)-mediated facilitation of the motor neuron pool (estimated using neuromuscular electrical stimulation with tendon vibration) were assessed before and after the same exercise intervention in 17 resistance trained men. Results showed decreases in peak plantar flexion torque (Experiment 1: -12.2%, Experiment 2: -16.9%), electrically evoked torque (20 Hz -15.3%, 80 Hz -15.3%, variable-frequency train -17.9%), and cSP (-3.8%; i.e., reduced inhibition) post-exercise which did not recover by 20 min. Electromyographic activity (EMG; -6%), corticospinal excitability (-9%), and PIC facilitation (-24.8%) were also reduced post-exercise but recovered by 10 min. Caffeine ingestion increased torque and EMG but did not notably affect corticospinal excitability, PIC amplification, or electrically evoked torque. The data indicate that a decrease in muscle function largely underpins the loss of force after repeated, high-intensity muscular efforts, but that the loss is exacerbated immediately after the exercise by simultaneous decreases in corticospinal excitability and PIC amplitudes at the motor neurons.

## New and Noteworthy

We provide evidence that impairment of persistent inward currents (PICs) at motoneurons could contribute to plantar flexor force loss immediately after high-intensity repeated contractions by showing a reduction in self-sustained torque elicited by tendon vibration. This impairment was recovered within 10 min, and prolonged (>20 min) post-exercise force loss was most clearly attributable to changes within the muscles themselves. Thus muscular, rather than neural, changes are most associated with prolonged fatigue.

## Introduction

The ability to perform repeated high-intensity muscular efforts, such as those involved in sprint- and strength training-type exercise, is limited by the accumulation of neuromuscular fatigue, where the work output (or force level) required to continue the activity can no longer be sustained. Fatigue can result from changes in function at multiple sites along the neuromuscular pathway (Bigland-Ritchie and Woods, 1984; Gandevia, 2001; Taylor and Gandevia, 2008), i.e., both central (i.e., neural) as well as peripheral (i.e., at the muscle and neuromuscular junction) mechanisms likely contribute to the functional loss. However, because of the inherent difficulties in simultaneously quantifying acute changes in multiple sites along the neuromuscular pathway, it has not been possible to piece together a comprehensive picture of relative, temporal changes.

At the central level, motor unit discharge rates slow during fatiguing voluntary contractions (Gandevia, 1998; Taylor et al., 2000b) and this reduction has been associated with numerous processes including changes in motor neuron intrinsic properties (Mendes, 2016), an increase in inhibitory input from sensory fibers (groups III and IV) (Gandevia, 1998), increases in cortical inhibition (Gruet et al., 2014), and suboptimal drive from the motor cortex (Taylor et al., 2000a; Gruet et al., 2014). While the precise influence of each process in this chain of events is unclear under the specific conditions of repeated, high-intensity, muscular efforts, another under explained process that might also impact motor unit discharge rates is the activation of persistent inward currents (PICs) at the motor neuron (Binder, 2003; Heckman et al., 2005). PICs are depolarizing currents generated by voltage-sensitive channels in the motor neuron that tend to remain active as long as the membrane potential remains above activation threshold (Heckman et al., 2005). PICs amplify and prolong synaptic input (Lee and Heckman, 1996; Hultborn et al., 2003; Heckman et al., 2008), allowing the motor neurons to fire more rapidly to produce high levels of muscular force (Hultborn et al., 2003). Given the importance of motor neuron PIC activation for force production in both maximal and submaximal contractions, a loss of the ability to generate PICs would reduce motor neuron excitability and increase the requirement for motor cortical output in order to maintain motor unit discharge rates and force output (McNeil et al., 2009), ultimately leading to a reduction in force and a higher level of perceived effort (Taylor et al., 2000b). However, it is still unclear whether fatigue caused by high-intensity intermittent contractions affects the ability to develop PICs as well as whether these changes are accompanied by changes in other neural sites.

At the peripheral level changes in excitation–contraction (E–C) coupling have been shown to contribute to the loss of force during fatiguing exercise (Allen et al., 1992; Pasquet et al., 2000) and are believed to be attributable to (1) changes in metabolite concentrations such as hydrogen ion (H^+^), inorganic phosphate (P_i_), extracellular potassium (K^+^), and adenosine diphosphate (ADP) (Allen et al., 2008; McKenna et al., 2008) and (2) changes in intracellular calcium handling, resulting from a reduction in sarcoplasmic calcium (Ca^2+^) release as well as reduced sensitivity of the contractile apparatus to Ca^2+^ (Allen et al., 2008), which ultimately impairs the E–C coupling process (Debold, 2016). Therefore, an understanding of the neuromuscular fatigue process requires critical evaluation of the effects of an exercise stimulus at both central and peripheral levels.

The manipulation of central and, possibly, peripheral physiology during fatiguing contractions is commonly performed through pharmaceutical interventions. Such manipulations may therefore not only prove practically beneficial for improving repeated-effort performance, but also provide important information relating to the physiological processes underpinning fatigue. Caffeine is one pharmaceutical agent that can influence multiple processes along the neuromuscular pathway and may therefore allow for a greater scrutiny of the impact of alterations in various processes. Importantly in the context of the current research, caffeine has been shown to increase the slope of the H-reflex recruitment curve and to increase the frequency of self-sustained motor unit firing (Walton et al., 2002). These observations strongly suggest that caffeine can increase motoneuronal gain and up regulate PIC amplitude, possibly through its positive effects on monoaminergic drive (Walton et al., 2002). Thus, caffeine may provide greater insight into the effects of monoaminergic drive on PIC-induced modulation of motor neuron excitability during fatiguing contractions.

Given the above, the main aim of the present study was to conduct a comprehensive examination of the mechanisms underpinning fatigue following repeated, high-intensity muscular efforts in trained individuals. To do this we performed a series of neuromuscular tests before and after fatiguing repeated plantar flexion contractions interspersed with brief rest to identify (1) the contribution of central vs. peripheral mechanisms to the ability to produce the required contraction force, (2) whether PIC-mediated facilitation of the motor neuron pool is impaired following the repeated high-intensity muscular contractions and whether any impairment persists into the post-exercise recovery period, and (3) whether caffeine would meaningfully affect physiological processes and thus allow greater scrutiny of the causes of functional loss. It was hypothesized that (1) both central and peripheral mechanisms would account for the loss in force following repeated fatiguing high-intensity muscular contractions, but that peripheral mechanisms would take longer to recover than central mechanisms, (2) PIC amplitude would be reduced following the muscular contractions but recover relatively quickly following the exercise, and (3) caffeine ingestion would attenuate the reduction in PIC amplitude during repeated high-intensity muscular contractions and minimize the loss of maximum force generating capacity.

## Materials and Methods

### Participants

Two experiments were conducted: 16 resistance-trained men volunteered to participate in Experiment 1 [age (mean ± SD), 25.8 ± 3.6 years; height, 1.71 ± 0.3 m; body mass, 92.3 ± 24.3 kg] and 17 volunteered for Experiment 2 (age, 25.5 ± 3.7 years; height, 1.76 ± 0.06 m; body mass, 86.6 ± 15.8 kg). Eight participants took part in both experiments. All participants completed a Physical Activity Readiness Questionnaire and a transcranial magnetic stimulation (TMS) Readiness Questionnaire, and reported no lower-limb neuromuscular disorders or contraindications to the safe use of TMS (i.e., no neurological disorders, medical implants, or use of medications that alter neuronal activity). The participants volunteered on the basis that they were involved in a lower-body resistance training program for muscular strength and/or power development for at least 12 months, had no known neurological or cardiovascular disease, and reported a minimal (<200 mg/day) caffeine consumption. They were required to abstain from taking any stimulants or depressants, including caffeine for at least 12 h and alcohol for at least 24 h, prior to testing and to refrain from performing sports or hard exercise training for 24 h prior to each experimental session.

The procedures performed during this research were approved by the Edith Cowan University Human Research Ethics Committee and were in agreement with the Declaration of Helsinki. Written informed consent was obtained from all participants.

### Study Design and Overview

Participants were thoroughly familiarized with tests prior to each experiment and were then tested in two sessions >72 h apart and at the same time of day (±1 h). In Session 1 of both experiments, each participant performed a high-intensity, intermittent series of muscular contractions of the plantar flexors of the right leg (i.e., the exercise protocol). The exercise was then precisely repeated in Session 2 where the exact number and intensity of muscle contractions was performed but where participants ingested 3 mg⋅kg^-1^ body mass of caffeine 1 h prior to trial commencement. Because we hypothesized that caffeine would reduce the rate of fatigue, the caffeine condition was always completed after the non-caffeine condition. Blinding of condition was not considered to be necessary since the aim of the trial was not to test the ergogenic effect of caffeine *per se*, but to examine the physiological responses to caffeine under a specific set of conditions. In Experiment 1, TMS was used to examine changes in corticospinal excitability and GABA_B_-mediated intracortical inhibition while in Experiment 2 a combined tendon vibration plus muscle stimulation (VIB+STIM) technique was used to estimate the motor neurons’ abilities to develop PICs (Trajano et al., 2014) and tetanic muscle contractions were evoked using different stimulation frequencies to probe the changes in muscle function.

Upon arrival at each testing session, the participant performed a standardized warm-up on a Monark cycle ergometer consisting of 5 min stationary cycling at 60 rpm with a 1-kg resistance. They were then seated on an isokinetic dynamometer (Biodex System 4 Pro, Biodex Medical System, Shirley, NY, United States) with the knee fully extended (0°) and ankle in the neutral position (0°; plane of foot relative to tibia) with the sole of foot perpendicular to the shank and the lateral malleolus of the fibula aligned with the dynamometer’s center of rotation. To minimize movement of the dynamometer system during maximal voluntary contractions (MVCs), the method used by Cannavan et al. (2012) was implemented. The seat was first positioned so that the knee angle was ∼30° with the leg relaxed, and then the leg was straightened so that the chair and dynamometer head were pressed apart, the mechanical compliance of the system reduced, and the leg acted like a strut with the muscles relaxed. Torque values were then expressed relative to the new baseline torque. The participant’s upper body, knee, and ankle were firmly secured to the dynamometer with straps and the contralateral leg rested on stool to avoid unwanted movements. Visual feedback of torque data was provided on a large monitor placed ∼2 m in front of the participant. Once seated, the participant performed five 3-s isometric plantar flexions ranging from 20 to 100% of perceived maximum effort with 30 s of rest between contractions as further warm-up.

### Fatiguing Exercise Protocol and Test Procedures

**FIGURE 1:**
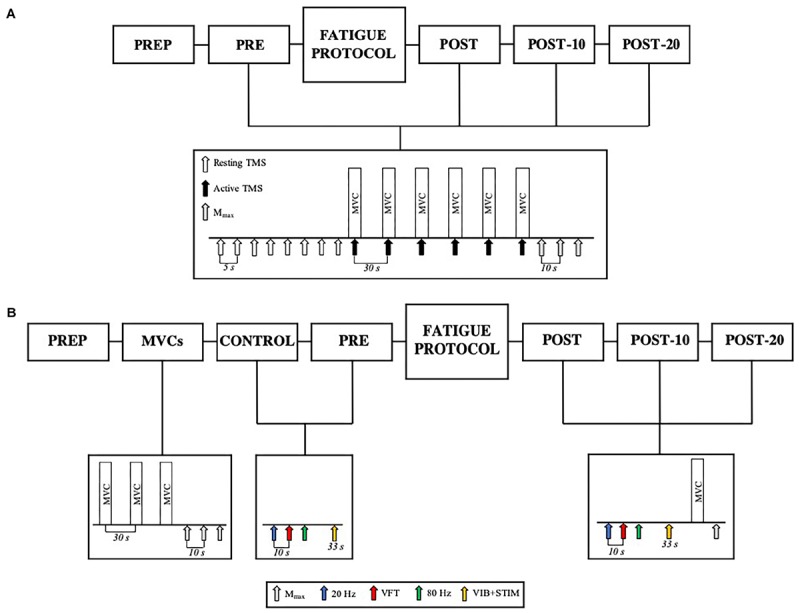
**(A)** Experiment 1 design. Participants completed PREP before being tested before (PRE) and immediately (POST), 10 min (POST-10) and 20 min (POST-20) after the fatiguing contractions. The test protocol consisted of eight resting TMS pulses (white arrows), six active TMS pulses (black arrows) superimposed during MVC (active TMS), and three resting *M*_max_ stimuli (gray arrows). **(B)** Participants completed PREP, followed by three MVCs and M-waves, Control, and PRE. Upon completion of the fatiguing exercise protocol, the participants were tested at POST, POST-10, and POST-20. The test protocol at Control and PRE consisted of 20 Hz (blue arrow), VFT (red arrow), 80 Hz (green arrow) stimulations, and the VIB+STIM protocol (yellow arrow). The POST test protocol consisted of 20 Hz, VFT, 80 Hz stimulations, VIB+STIM, one MVC, and one *M*_max_ measurement.

The fatiguing exercise protocol consisted of six sets of isometric plantar flexion contractions (3-s contraction/2-s rest) reaching a target level of 85% MVC with a 90-s rest between sets. Each set of contractions was completed when the required torque level could not be attained in two consecutive repetitions, i.e., each set contained a different number of contractions, with fewer repetitions performed in the latter sets of the protocol. This protocol was utilized to mimic resistance training practices of performing sets of muscle contractions until volitional failure. In Experiment 1, a test protocol of eight resting TMS pulses each separated by 5 s (described in detail below), six 3-s MVCs with superimposed TMS pulses separated by 30 s, and three tibial nerve stimulations to evoke M-waves (*M*_max_) were completed 180 s before (PRE) and immediately (POST), 10 (POST-10) and 20 min (POST-20) after completing the exercise protocol (see Figure 1 for details). In Experiment 2, electrically evoked torque under three train stimulation conditions as well as VIB+STIM measurements were performed both 7 (within-day control; CON) and 2 min (PRE) prior to the repetition of the same exercise protocol performed in Experiment 1 (Figure 1). Upon completion of the exercise protocol, electrically evoked torque, VIB+STIM, MVC, and *M*_max_ measurements were performed. These were again repeated 10 (POST-10) and 20 min (POST-20) after the fatiguing exercise protocol. Participants were instructed to perform the MVCs with the greatest possible rate of force development, to hold for 3 s (indicated by the experimenter) and then to relax as quickly as possible. Participants received strong verbal encouragement and visual feedback of performance from each MVC completed. In Experiment 1, maximal voluntary isometric plantar flexion torque from the six MVCs at each time point was averaged and used as a measure of voluntary force production. No statistical difference was observed for changes in torque between the average of six-, peak-, or median MVCs (χ^2^_(2,_ *_N_*_=__96)_ = 1.000, *p* = 0.607). Therefore, the average was chosen to better compare the changes in torque with changes in motor-evoked potentials (MEPs). In Experiment 2, the best score from the three MVCs at PRE was recorded and compared to the single MVC performed at all other time points. All data were recorded synchronously at 2,000 Hz on a personal computer running LabChart software (version 8.1.5, ADInstruments, NSW, Australia) using a 16-bit analog-to-digital converter (PowerLab 16/35, ADInstruments, NSW, Australia).

### Transcranial Magnetic Stimulation (TMS) Procedure (Experiment 1)

Magnetic stimuli were delivered to the contralateral (i.e., left) primary motor cortex by a double-cone coil (110 mm diameter) attached to a Magstim 200^2^ stimulator. The coil was oriented to induce a posterior–anterior current. To find the optimum stimulation site (hotspot), the coil was moved over the motor cortex area contralateral to the plantar flexors of the right leg in 1-cm increments laterally, anteriorly, and posteriorly to the vertex using a pre-defined grid marked on a cap worn by participants which was set according to the intersection of lines between the inion and nasion and the left and right ear tragus. The position that elicited the greatest average of three soleus (SOL) MEPs while evoking a minimal tibialis anterior (TA) response (<50% of SOL MEP) was marked on the cap to ensure that accurate positioning was maintained. A custom-made coil holder and an experienced investigator maintained coil placement throughout the experiments. Using Parameter Estimation by Sequential Testing (Awiszus, 2003) procedures through the TMS Motor Threshold Assessment Tool software (MTAT 2.0) (Awiszus and Borckardt, 2011; Silbert et al., 2013), resting motor threshold was determined as the lowest TMS intensity that yielded a peak-to-peak resting SOL MEP amplitude of at least 60 μV (mean ± SD 55.7 ± 10.5% of maximal stimulator output). TMS intensity was then set at 120% of resting motor threshold (62.7 ± 19.9% of maximal stimulator output) and this intensity was kept constant throughout the session (Soto et al., 2006).

### Tibial Nerve Stimulation (Experiments 1 and 2)

Percutaneous tibial nerve stimulation was used to evoke M-waves to assess potential changes in muscle action potential propagation as well as to normalize both MEP and electromyogram (EMG) measurements, as described below. The stimulation intensity required to evoke the maximal M-wave amplitude (*M*_max_) was determined by delivering single 0.2-ms square-wave pulses to the tibial nerve of the right leg using a constant-current stimulator (DS7H, Digitimer Ltd., Welwyn Garden City, United Kingdom). The cathode electrode (pick-up area 77 mm^2^; Unilect 4535M, Ag/AgCl, Unomedical Ltd., Redditch, United Kingdom) was positioned over the tibial nerve in the popliteal fossa and the anode (5 × 9 cm; Dura-Stick Plus, DJO Global LLC., Vista, CA, United States) was positioned proximal to the patella. An elastic band was placed around the knee over the cathode to hold it in a constant position and to apply constant pressure throughout testing sessions. The stimulation site that evoked the greatest SOL M-wave response at a submaximal stimulation intensity was located by a hand-held cathode electrode pen (Compex Motor Point Pen, DJO Global LLC., CA, United States). The stimulation intensity for *M*_max_ was determined by increasing stimulator intensity in 5–10 mA increments from a sub-motor threshold intensity until the M-wave for both SOL and MG amplitudes plateaued. Ten-second intervals were imposed between stimuli and the participants were at rest in the isokinetic dynamometer. The greatest stimulus intensity required to find SOL or MG *M*_max_ was then increased by 50% to account for activity-dependent axonal hyperpolarization after fatigue (Vagg et al., 1998). The supramaximal stimulus intensity for *M*_max_ was held constant throughout the experiment.

### Muscle Activity (EMG) (Experiments 1 and 2)

Surface EMG was recorded during voluntary contractions and TMS procedures. Voluntary EMG was recorded from SOL, medial gastrocnemius (MG), and TA of the right leg using bipolar configurations of two Ag/AgCl self-adhesive electrodes (inter-electrode distance of 2 cm; Blue Sensor N-00-S, 28 mm^2^, Ambu, Ballerup, Denmark). SOL EMG location was ∼3 cm below the distal head of MG. MG EMG was positioned over the most prominent area of MG when isometrically contracted, and TA EMG was placed one-third of the distance between the lateral epicondyle of the tibia and the medial malleolus. To obtain clearer evoked responses (i.e., MEP, cSP, and *M*_max_ data), a pseudo-monopolar EMG configuration was also used over SOL (SOL_PSU_). This pseudo-monopolar configuration provides a better representation of the electrical characteristics of the action potentials, compared with the bipolar (single-differential) where part of the electrical signal is lost due to phase cancelation (Rodriguez-Falces and Place, 2018). The active electrode was positioned just medial to the SOL bipolar EMG electrodes and the dispersive electrode ∼3 cm superior to the medial malleolus and over the Achilles tendon–soleus muscle–tendon junction (Blazevich et al., 2012). The reference electrodes were positioned on the medial and lateral malleoli of the ankle. The skin beneath the electrodes was shaved, abraded, and cleaned with alcohol to reduce inter-electrode resistance below 5 kΩ. EMG data were sampled at a 2,000-Hz analog–digital conversion rate using a Dual BioAmp EMG System (ADInstruments, NSW, Australia) and band-pass filtered (20–500 Hz) using LabChart Software (PowerLab System, ADInstruments, v. 8.1.5, NSW, Australia). SOL, MG, and TA muscle activities during plantar flexion were expressed as root mean square EMG amplitudes using a symmetric 500-ms averaging window and SOL and MG EMG were normalized to *M*_max_ amplitude (EMG/M) (Kalmar and Cafarelli, 2004b). Percent voluntary activation (using interpolated twitch technique) was not used in the present study because (1) only a limited number of tests could be performed at each timepoint and (2) while it provides an estimate of the relative activation of the muscle it is influenced by a number of factors in addition to the central drive to the muscle (De Haan et al., 2009; Neyroud et al., 2016). In addition, both SOL/M and MG/M were averaged to obtain a global measure of triceps surae EMG (TS/M) (Trajano et al., 2013). To measure MEP and *M*_max_ amplitudes, EMG data remained unfiltered and the peak-to-peak amplitude was used. The TMS-induced EMG cortical silent period (cSP) duration was determined by visual inspection of the SOL_PSU_ EMG records and measured from the onset of the MEP to the return of voluntary EMG activity after the silent period (Damron et al., 2008; Säisänen et al., 2008). Changes in MEP/M ratio and cSP were used to assess corticospinal excitability and GABA_B_-mediated intracortical inhibition, respectively.

### Muscle Stimulation Procedures (Experiment 2)

A constant-current electrical stimulator (DS7, Digitimer Ltd., Welwyn Garden City, United Kingdom) was used to deliver an electrical square-wave stimulus (0.5-ms pulse width) to the plantar flexor muscle belly through two self-adhesive electrodes (5 × 9 cm; Dura-Stick Plus, DJO Global LLC., Vista, CA, United States). The cathode was placed on the medial (GM) and lateral (GL) gastrocnemius muscle bellies (superior to the MG electrodes) where the greatest motor response was elicited (i.e., assumed motor point) and the anode was placed over the distal SOL myotendinous junction (inferior to the MG electrodes). Two elastic bands were placed around the calf, one over each electrode, to apply a constant pressure throughout testing sessions.

For all tetanic stimulation trains, the intensity necessary to reach 50% of MVC with a 0.5-s 80-Hz tetanic stimulation was used (Martin et al., 2004). Three tetanic stimulations of the same duration (0.5 s) were delivered to test for E–C coupling efficiency: (1) 20 Hz train; (2) variable frequency train (VFT) (i.e., 20 Hz train with the first two pulses at 100 Hz); and (3) 80 Hz train. The peak torque produced by the 20- and 80-Hz stimulations were used to calculate the 20:80 Hz torque ratio, which was used as a measure of E–C coupling efficiency, specifically Ca^2+^ release through the ryanodine receptor (Martin et al., 2004; Balog, 2010). The ratio of the torques evoked by the variable vs. constant frequency trains (20:VFT) was used to estimate the muscle’s capacity to utilize a high-frequency double discharge at contraction onset, which is hypothesized to be strongly affected by Ca^2+^ sensitivity of the muscle fibers (Burke et al., 1970; Binder-Macleod and Kesar, 2005).

### Motor Neuron Facilitation (Tendon Vibration With Superimposed Neuromuscular Electrical Stimulation) (Experiment 2)

**FIGURE 2:**
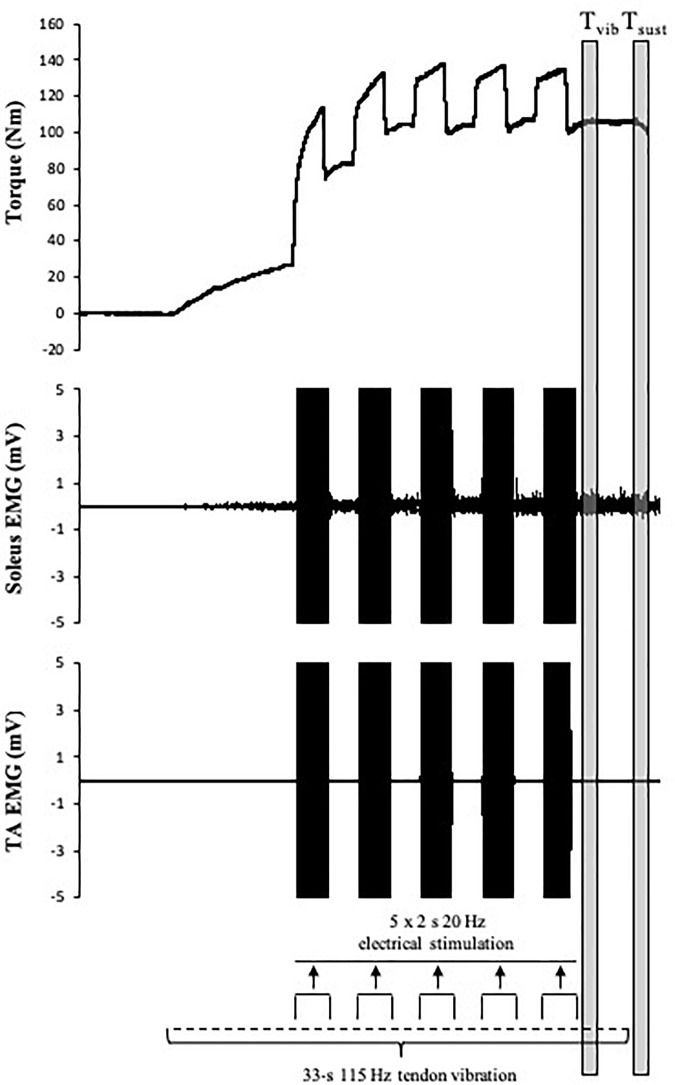
Schematic representation of the tendon vibration and superimposed tibial nerve stimulation protocol used to elicit reflexive muscular contractions and the respective time points at which torque was recorded. *T*_vib_, torque is measured after the fifth (last) bout of electrical stimulation; *T*_sust_, torque is measured 500 ms after vibration cessation (self-sustained torque). EMG trace indicates pronounced SOL but minimal TA muscle activity throughout the VIB+STIM sequence.

A combined tendon vibration plus muscle stimulation (VIB+STIM) technique was used to estimate PIC magnitude (Trajano et al., 2014). This protocol has been shown to display PIC-like features such as joint angle dependence (i.e., affected by reciprocal inhibition), increases after repetitive activation (i.e., wind-up effect), self-sustained motor unit firing, and the inhibition of sustained firing by voluntary antagonist muscle activation (Trajano et al., 2014). With the participant’s ankle in a slightly dorsiflexed position (-10°), the Achilles tendon of the right leg was mechanically vibrated at 115 Hz (1-mm deflections) by a hand-held vibrator (Vibrasens, Techno Concepts, Mane, France). The vibrator was held with steady pressure against a marked point in line with the medial malleolus on the Achilles tendon for 33 s. After the first 10 s of vibration, five 2-s bursts of neuromuscular electrical stimulation at a frequency of 20 Hz were superimposed on the ongoing tendon vibration at 4-s intervals (Figure 2). The experimental setup for muscle stimulation was the same as that used to measure E–C coupling efficiency (described above). For all electrical stimulations, an intensity that elicited a torque response of 20% MVC with a 0.5-s 20 Hz tetanic stimulation was used. During the VIB+STIM protocol, participants were instructed to hold onto the shoulder straps and not voluntarily activate the plantar or dorsiflexor muscles. The left leg was positioned on a stool so that both legs were straight which prevented any extraneous movement throughout the test. To further reduce the chances of voluntary calf activation, participants were asked to count in 2 s from zero (i.e., 0, 2, 4, 6, etc.) at a self-selected pace throughout the VIB+STIM protocol. Evoked torque from the VIB+STIM protocol (Figure 2) was analyzed as the mean torque in a 1-s window at two different time points during the protocol: (1) during vibration starting 500 ms after completion of the fifth (last) burst of electrical stimulation (i.e., torque during vibration; *T*_vib_) and (2) 500 ms after the vibration was ceased (sustained torque; *T*_sust_). As plantar flexor muscles impose a small passive torque even when the muscle is relaxed, *T*_vib_ and *T*_sust_ torque values were normalized and presented as changes from the baseline (resting) value. Response to vibration occurred in 17 of 25 participants tested as determined by a visible wind-up during and self-sustained torque following the VIB+STIM sequence. These “responders” thus formed the study cohort.

### Statistical Analysis

Prior to analysis, the data were assessed for normality of distribution, homogeneity of variance (Levene’s test), and sphericity (Mauchly’s test). In Experiment 1, separate two-way repeated measures ANOVAs were performed to compare changes in voluntary torque, MEP, MEP/M ratio, cSP duration, *M*_max_, and TA variables between non-caffeine (NON-CAF) and caffeine (CAF) conditions and over time (PRE, POST, POST-10, and POST-20). A two-way repeated measures MANOVA was performed to compare changes in SOL/M_,_ MG/M and TS/M between conditions and over time. In Experiment 2, separate two-way repeated measures ANOVAs were performed to compare changes in voluntary torque, *M*_max_, and TA variables between conditions (NON-CAF vs. CAF) and over time (PRE, POST, POST-10, and POST-20). Two-way repeated measures MANOVAs were performed to compare changes in SOL/M, MG/M, and TS/M, as well as evoked torque from 20 Hz, 80 Hz and VFTs, 20:80 Hz and 20:VFT ratios, and *T*_vib_, *T*_sust_ between conditions and over time. Where statistical significance was detected, *post hoc* analysis was conducted using Fisher’s LSD test. Statistical analysis was performed using SPSS version 24.0 (SPSS Inc., Chicago, IL, United States). All data are reported as mean ± SD in text (and mean ± SE in graphs), confidence intervals (95%), and Hedges’ *g* effect sizes. Statistical significance was accepted at an α-level of 0.05.

## Results

### Experiment 1: The Effect of Fatigue and Recovery From Repeated High-Intensity Muscle Contractions on Cortico-Motoneuronal Pathway Efficiency

To minimize repetition of results, time effects are reported as pooled NON-CAF and CAF.

**FIGURE 3:**
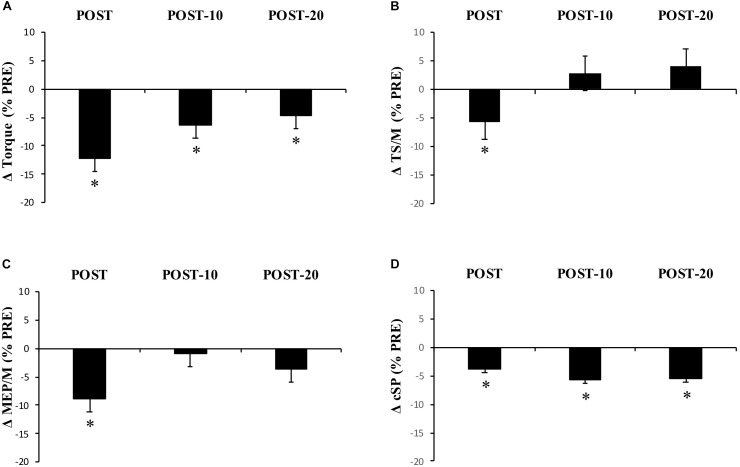
Experiment 1, changes in torque, EMG, MEP/M, and cSP measured from PRE to immediately (POST), 10 min (POST-10), and 20 min (POST-20) after exercise. Changes in **(A)** MVC torque, **(B)** triceps surae EMG normalized to M-wave amplitude (EMG/M), **(C)** motor-evoked potential amplitude during MVCs normalized to M-wave amplitude (MEP/M), and **(D)** cortical silent period (cSP). ^∗^Significantly different from PRE, *p* < 0.05.

**FIGURE 4:**
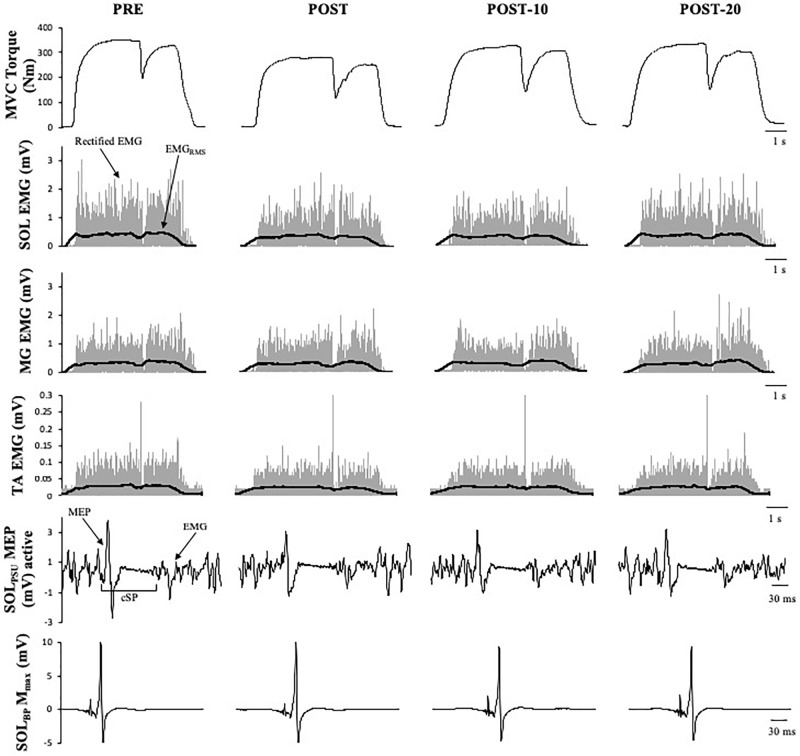
Example data obtained from one participant in the non-caffeine session of Experiment 1 before (PRE) and immediately (POST), 10 min (POST-10) and 20 min (POST-20) after the exercise protocol. Decreases in MVC torque, SOL_RMS_, and MEP amplitude during MVC (second row) and are noticeable immediately after the exercise protocol.

Maximum voluntary torque – there were significant time (*F*_(2.169,15)_ = 34.391, *p* < 0.001) (Figure 3) and condition (*F*_(1,15)_ = 10.843, *p* = 0.005) effects for maximum plantar flexion torque. There was a decrease in maximum voluntary torque at POST [-12.2 ± 6.5% (CI = -22, -3%), *p* < 0.001, *g* = 0.642] which did not recover by POST-10 [-6.3 ± 6.1% (-16, 4%), *p* < 0.001, *g* = 0.315] or POST-20 [-4.8 ± 6.5% (-15, 5%), *p* = 0.002, *g* = 0.239). The ingestion of caffeine resulted in a greater torque output when compared to the non-caffeine condition (Figure 2); however, no interaction effect (*F*_(2.655,15)_ = 1.489, *p* = 0.235) was observed, indicating a lack of difference in the change in torque over time between conditions. An example of raw data is provided in Figure 4.

The number of repetitions performed throughout the exercise protocol did not statistically differ between conditions (*p* = 0.908). Of the 16 participants, 9 replicated exactly the number of repetitions performed in the non-caffeine session, 4 performed one less repetition, 2 performed two fewer repetitions, and 1 performed six fewer repetitions in total.

Muscle activity (EMG) – there were significant time effects for maximal SOL/M (*F*_(3,15)_ = 6.233, *p* = 0.001) and TS/M (*F*_(3,15)_ = 9.505, *p* < 0.001) (Figure 3) during MVCs with pooled data showing decreases of -7.6 ± 14.9% [(-38, 22%), *p* = 0.003, *g* = 0.186] and -5.8 ± 12% [(-21, 8%), *p* = 0.012, *g* = 0.226] immediately after exercise, respectively. While a main effect was observed for MG/M (*F*_(3,15)_ = 8.086, *p* < 0.001), the change immediately after exercise did not reach statistical significance [-3.9 ± 14.3% (-25, 14%), *p* = 0.076, *g* = 0.205). All EMG amplitudes returned to PRE levels by 10 min (Table 1). There were no significant changes in TA EMG during MVCs. Caffeine ingestion was associated with a greater MG/M (*F*_(1,15)_ = 7.581, *p* = 0.015) and TS/M (*F*_(1,15)_ = 11.702, *p* = 0.004); however, no condition-by-time interaction effect was observed [MG/M (*F*_(3,15)_ = 1.628), *p* = 0.196) and TS/M (*F*_(3,15)_ = 1.115, *p* = 0.353)] indicating that caffeine did not cause a change in muscle activity over time between the conditions.

**Table 1:** Plantar flexor torque, EMG, and TMS data measured in Experiment 1.

	Exercise condition	PRE	POST	POST-10	POST-20
Torque (Nm)	NON-CAF	214.5 ± 41.1	186.9 ± 35.8^a^	197.9 ± 41.1^a^	201.6 ± 43.2^a^
	CAF^∗^	222.1 ± 45.4	196.1 ± 41.2^a^	211.3 ± 43.5^a^	214.1 ± 41.8^a^
SOL/M	NON-CAF	0.030 ± 0.015	0.028 ± 0.014	0.031 ± 0.013	0.031 ± 0.013
	CAF	0.033 ± 0.011	0.030 ± 0.011	0.033 ± 0.011	0.033 ± 0.011
MG/M	NON-CAF	0.033 ± 0.009	0.033 ± 0.009	0.035 ± 0.010	0.035 ± 0.009
	CAF^∗^	0.039 ± 0.010	0.036 ± 0.008	0.040 ± 0.009	0.042 ± 0.009
TS/M	NON-CAF	0.033 ± 0.011	0.031 ± 0.011	0.033 ± 0.010	0.033 ± 0.010
	CAF^∗^	0.036 ± 0.009	0.034 ± 0.008	0.037 ± 0.009	0.038 ± 0.009
TA (mv)	NON-CAF	0.107 ± 0.040	0.096 ± 0.039	0.101 ± 0.036	0.095 ± 0.040
	CAF	0.113 ± 0.042	0.106 ± 0.051	0.110 ± 0.051	0.095 ± 0.039
MEP/M	NON-CAF	0.231 ± 0.093	0.207 ± 0.073	0.220 ± 0.069	0.216 ± 0.080
	CAF	0.235 ± 0.085	0.209 ± 0.093	0.227 ± 0.087	0.222 ± 0.080
MEP_rest_/M	NON-CAF	0.053 ± 0.029	0.067 ± 0.034	0.061 ± 0.039	0.068 ± 0.057
	CAF	0.054 ± 0.030	0.065 ± 0.046	0.059 ± 0.034	0.057 ± 0.034
cSP (ms)	NON-CAF	105.7 ± 15.7	100.4 ± 12.5^a^	99.8 ± 13.8^a^	101.3 ± 12.6^a^
	CAF	105.3 ± 17.1	100.5 ± 12.8^a^	97.8 ± 14.2^a^	96.2 ± 12.3^a^
SOL_BP_ *M*_max_	NON-CAF	7.117 ± 3.102	6.649 ± 3.102^a^	6.252 ± 2.928^a^	6.259 ± 2.888^a^
	CAF^∗^	6.135 ± 2.833	5.763 ± 2.638	5.461 ± 2.569^a^	5.511 ± 2.599^a^
MG *M*_max_	NON-CAF	12.732 ± 3.586	11.680 ± 3.462^a^	11.171 ± 3.465^a^	11.257 ± 3.404^a^
	CAF	12.999 ± 3.181	12.086 ± 3.226^a^	11.192 ± 3.176^a^	10.939 ± 3.165^a^
SOL_PSU_ *M*_max_	NON-CAF	21.607 ± 5.603	20.910 ± 5.107	20.182 ± 4.628	20.080 ± 4.686
	CAF	21.476 ± 4.447	21.613 ± 4.481	20.593 ± 4.038	20.584 ± 4.280

*Values are mean ± SD. Torque, mean torque during maximal voluntary isometric plantar flexion. SOL/M, MG/M, TS/M, soleus, medial gastrocnemius, and triceps surae (SOL + MG) root-mean square EMG normalized to maximal M-wave amplitude; TA, tibialis anterior root-mean square EMG; MEP/M, motor-evoked potential amplitude during MVC normalized to M_max_; MEP_rest_/M, motor-evoked potential amplitude during rest normalized to M_max_; SOL_BP_ M_max_, soleus bipolar EMG; MG M_max_, medial gastrocnemius EMG; SOL_PSU_ M_max_, soleus pseudo-monopolar EMG. ^∗^Between-group effect, significantly different from NON-CAF, p < 0.05; ^a^Significant time (but no condition × time) effect, p < 0.05.*

MEP amplitude – there was a significant time effect for MEP/M measured during MVC (*F*_(3,15)_ = 4.339, *p* = 0.009) (Figure 3), which decreased at POST [-8.8 ± 20.8% (-29, 8%), *p* = 0.01, *g* = 0.281] but recovered by 10 min [-0.9 ± 19.6% (-22, 14%), *p* = 0.184, *g* = 0.108]. However, when measured at rest (MEP_rest_/M) no time (*F*_(3,15)_ = 1.807, *p* = 0.182), condition (*F*_(1,15)_ = 0.222, *p* = 0.645), or condition-by-time interaction (*F*_(2.028,15)_ = 1.356, *p* = 0.275) effects were observed.

Cortical silent period – there was a significant time effect for cSP (*F*_(2.204,15)_ = 7.189, *p* = 0.002) (Figure 3) in which a significant decrease of -3.8 ± 10.2% [(-11, 2%), *p* = 0.016, *g* = 0.341) was observed immediately after exercise, which remained at POST-10 [-5.7 ± 9.2% (-13, 1%), *p* = 0.004, *g* = 0.433] and POST-20 [-5.6 ± 9.6% (-13, 1%), *p* = 0.004, *g* = 0.456].

M-wave amplitude – there were significant time effects for SOL *M*_max_ (*F*_(3,15)_ = 18.919, *p* < 0.001) and MG *M*_max_ (*F*_(1.698,15)_ = 53.563, *p* < 0.001) with decreases of -6.7 ± 9.6% [(-29, 16%), *p* = 0.01, *g* = 0.140] in SOL *M*_max_ and -8 ± 4.7% [(-21, 5%), *p* = <0.001, *g* = 0.288] in MG *M*_max_ immediately after exercise. M-wave amplitude did not return to PRE levels by 20 min (Table 1).

### Experiment 2 – Reflexive Torque (Motor Neuron Facilitation) and Muscle-Evoked Responses (Ca^2+^ Kinetics)

**FIGURE 5:**
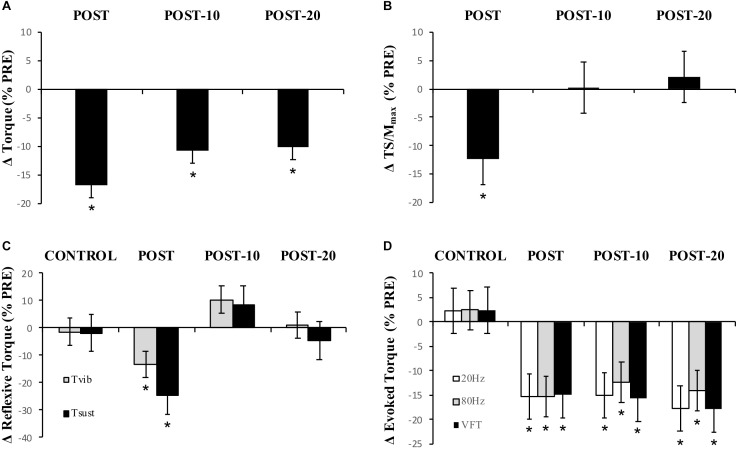
Experiment 2, changes in torque, EMG, reflexive torque, and tetanic torque measured from before to immediately (POST), 10 min (POST-10) and 20 min (POST-20) post-exercise. **(A)** MVC torque, **(B)** triceps surae EMG normalized to M-wave (TS/*M*_max_), **(C)** mean torque after the fifth (last) stimulation during vibration (*T*_vib_) and self-sustained torque (*T*_sust_) measured 500 ms after vibration cessation, **(D)** peak torque during 20-Hz, 80-Hz, and VFT trains of electrical stimulation. ^∗^Significantly different from PRE, *p* < 0.05.

**FIGURE 6:**
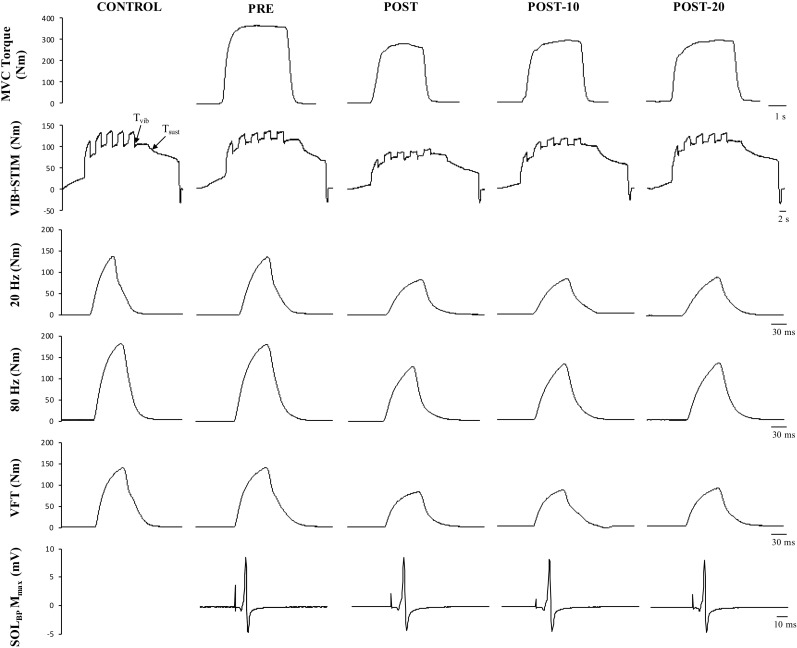
Example data obtained from one participant in the non-caffeine session in Experiment 2 before (PRE) and immediately (POST), 10 min (POST-10) and 20 min (POST-20) after the exercise protocol. Decreases in MVC torque (first row), reflexive torque (second row), and tetanic stimulations are noticeable immediately after the exercise protocol.

Maximum voluntary torque – similar to Experiment 1, there were significant time (*F*_(1.596,16)_ = 51.016, *p* < 0.001) (Figure 5) and condition (*F*_(1,16)_ = 8.529, *p* = 0.011) (Figure 5) effects for torque; however, no interaction effect (*F*_(2.219,16)_ = 2.985, *p* = 0.059) was observed (again pooled data are presented herein to minimize repetition, but values in NON-CAF and CAF conditions are separately reported in Table 2). Torque decreased at POST [-16.9 ± 7.3% (-25, -8%), *p* < 0.001, *g* = 0.925] and did not recover by POST-10 [-10.8 ± 7.5% (-19, -2%), *p* < 0.001, *g* = 0.604] or POST-20 [-10.2 ± 8% (-19, -1%), *p* < 0.001, *g* = 0.545]. The ingestion of caffeine resulted in a greater torque output when compared to the non-caffeine condition but did not affect force production at any specific time point, i.e., before or in recovery from exercise (lack of condition-by-time interaction effect). An example of raw data is provided in Figure 6.

**Table 2:** Plantar flexor torque, EMG, reflexive torque, and electrically evoked torque data measured in Experiment 2.

	Exercise condition	CONTROL	PRE	POST	POST-10	POST-20
Torque (Nm)	NON-CAF	N/A	244.3 ± 47	203.8 ± 40.5^a^	220 ± 43.9^a^	214.9 ± 46.1^a^
	CAF^∗^	N/A	254.6 ± 44.6	213.5 ± 41.4^a^	224.9 ± 40.6^a^	233.6 ± 42.5^a^
SOL/M	NON-CAF	N/A	0.041 ± 0.017	0.040 ± 0.018	0.043 ± 0.019	0.042 ± 0.028
	CAF	N/A	0.041 ± 0.016	0.036 ± 0.016	0.043 ± 0.021	0.043 ± 0.019
MG/M	NON-CAF	N/A	0.039 ± 0.014	0.033 ± 0.012^a^	0.038 ± 0.011	0.038 ± 0.013
	CAF^∗^	N/A	0.042 ± 0.015	0.035 ± 0.011^a^	0.040 ± 0.014	0.044 ± 0.014
TS/M	NON-CAF	N/A	0.037 ± 0.011	0.033 ± 0.010^a^	0.037 ± 0.009	0.037 ± 0.012
	CAF^∗^	N/A	0.041 ± 0.014	0.034 ± 0.010^a^	0.039 ± 0.012	0.043 ± 0.013
TA (mv)	NON-CAF	N/A	0.074 ± 0.029	0.071 ± 0.037	0.079 ± 0.042	0.073 ± 0.037
	CAF	N/A	0.116 ± 0.115	0.087 ± 0.078	0.086 ± 0.074	0.089 ± 0.079
*T*_vib_ (Nm)	NON-CAF	24.5 ± 23.5	30.4 ± 25.6	26 ± 22.9	22.1 ± 27.4	31.2 ± 29.1
	CAF	31.8 ± 31.4	30.9 ± 30.6	22.1 ± 27.4^a^	31 ± 28.1	30.1 ± 25.3
*T*_sust_ (Nm)	NON-CAF	23.6 ± 24.7	30.3 ± 26.2	24.6 ± 22.4	31.5 ± 27.4	29.7 ± 25.2
	CAF	32.2 ± 32.2	32.1 ± 30.6	20.5 ± 26.2^a^	30.5 ± 28.5	28.5 ± 31.4
20 Hz (Nm)	NON-CAF	91.8 ± 17.4	90 ± 17.8	75.7 ± 24^a^	75 ± 26^a^	72.8 ± 26.6^a^
	CAF	96.4 ± 16.8	94.3 ± 14.9	81.1 ± 16.6^a^	81.7 ± 11.6^a^	79.5 ± 13.5^a^
80 Hz (Nm)	NON-CAF	121.5 ± 25.5	118.5 ± 26.9	100.5 ± 32.3^a^	103.1 ± 36.9^a^	101.7 ± 36.9^a^
	CAF	126.8 ± 22.9	124.5 ± 20.4	106.7 ± 20.2^a^	110.6 ± 14.9^a^	109.1 ± 17.9^a^
VFT (Nm)	NON-CAF	93.3 ± 18.5	91 ± 18.9	77.3 ± 24.5^a^	75.4 ± 26.9^a^	74.2 ± 27.3^a^
	CAF	98.3 ± 17.3	96.4 ± 15.3	82.6 ± 16.3^a^	82.9 ± 11.8^a^	80.7 ± 13.4^a^
20:80 Hz ratio	NON-CAF	0.761 ± 0.056	0.767 ± 0.057	0.753 ± 0.052	0.731 ± 0.076	0.723 ± 0.073
	CAF	0.762 ± 0.047	0.760 ± 0.054	0.761 ± 0.061	0.739 ± 0.048	0.730 ± 0.049
20:VFT ratio	NON-CAF	0.986 ± 0.024	0.991 ± 0.023	0.977 ± 0.024	0.999 ± 0.062	0.988 ± 0.039
	CAF	0.981 ± 0.026	0.979 ± 0.025	0.981 ± 0.021	0.986 ± 0.035	0.985 ± 0.022
SOL_BP_ *M*_max_	NON-CAF	N/A	6.144 ± 3.362	4.724 ± 2.690^a^	4.605 ± 2.657^a^	4.943 ± 3.158^a^
	CAF	N/A	5.453 ± 1.860	4.350 ± 1.57^a^	4.198 ± 1.608^a^	4.224 ± 1.711^a^
MG *M*_max_	NON-CAF	N/A	13.424 ± 3.637	12.543 ± 3.241	11.466 ± 2.869^a^	11.688 ± 2.478^a^
	CAF	N/A	13.011 ± 3.661	11.673 ± 2.251	11.126 ± 2.778^a^	10.915 ± 2.849^a^

*Values are mean ± SD. Torque, peak torque during maximal voluntary isometric plantar flexion. SOL/M, MG/M, TS/M, soleus, medial gastrocnemius, and triceps surae (SOL+MG) root-mean square EMG normalized to maximal M-wave amplitude; TA, tibialis anterior root-mean square EMG; T_vib_, T_sust_, mean torque after the fifth (last) stimulation during vibration and self-sustained torque 500 ms after vibration; 20 Hz, 80 Hz, VFT (variable frequency train) tetanic torque values during stimulation trains; 20:80 Hz, 20:VFT, stimulation train ratios; SOL_BP_ M_max_, soleus bipolar EMG; MG M_max_, medial gastrocnemius EMG. ^∗^Between-group effect, significantly different from NON-CAF, p < 0.05. ^a^significant time (but no condition × time) effect, p < 0.05.*

The number of repetitions performed throughout the exercise protocol did not statistically differ between conditions (*p* = 0.778). Of the 17 participants, 10 replicated exactly the number of repetitions performed in the non-caffeine session, while 5, 1, and 1 participants failed by 2, 3, and 5 repetitions, respectively.

Muscle activity (EMG) – there were significant time effects for maximal MG/M (*F*_(1.815,15)_ = 8.88, *p* < 0.002) and TS/M (*F*_(1.992,16)_ = 12.171, *p* < 0.001) (Figure 5) during MVCs with decreases of -11.7 ± 23.4% [(-37, 0%), *p* = 0.009, *g* = 0.469] and -12.3 ± 18.2% [(-28, -1%], *p* = 0.01, *g* = 0.493] immediately after exercise, respectively. In SOL/M, the trend toward a time effect did not reach statistical significance (*F*_(3,16)_ = 2.385, *p* = 0.083). All EMG amplitudes returned to PRE levels by 10 min (Table 2). There were no significant changes in TA EMG during MVCs. Caffeine ingestion was associated with a greater MG/M (*F*_(1,16)_ = 8.399, *p* = 0.012) and TS/M (*F*_(1,16)_ = 8.971, *p* = 0.010); however, no condition-by-time interaction effect was observed for MG/M (*F*_(3,16)_= 1.748, *p* = 0.172) and TS/M (*F*_(1.805,16)_ = 3.427, *p* = 0.053), indicating that caffeine did not cause a change in muscle activity over time between the conditions (Figure 5). No condition effect was observed in SOL/M (*F*_(1,16)_ = 0.012, *p* = 0.914).

Reflexive torque – there was a significant time effect for both *T*_vib_ (*F*_(3,16)_ = 3.803, *p* = 0.016) and *T*_sust_ (*F*_(3,16)_ = 3.622, *p* = 0.021) (Figure 5), with decreases of -13.2 ± 60.5% [(-70, 23%), *p* = 0.047, *g* = 0.243] and -24.8 ± 53.6% [(-69, 14%), *p* = 0.026, *g* = 0.322], respectively, immediately after exercise. However, these variables recovered by 10 min post-exercise. No difference was detected between CON and PRE for *T*_vib_ [-1.4 ± 36.5% (-57, 39%), *p* = 0.204, *g* = 0.090] or *T*_sust_ [-1.8 ± 43.7% (-55, 34%), *p* = 0.139, *g* = 0.114], suggesting that the measures were reliable. No condition effects were noted for *T*_vib_ (*F*_(1,16)_ = 0.736, *p* = 0.404) or *T*_sust_ (*F*_(1,16)_ = 0.338, *p* = 0.916).

Muscle evoked responses – there were significant time effects for torque evoked in trains of 20 Hz (*F*_(1.487,16)_ = 21.867, *p* < 0.001), 80 Hz (*F*_(1.653,16)_ = 24.756, *p* < 0.001), and VFT (*F*_(1.524,16)_ = 23.480, *p* < 0.001) (Figure 5), with decreases of -15.3 ± 16.1% [(-24, -5%), *p* < 0.001, *g* = 0.725], -15.3 ± 12.9% [(-24, -4%), *p* < 0.001, *g* = 0.689], and -14.9 ± 15.6% [(-24, -5%], *p* < 0.001, *g* = 0.701] immediately after exercise, respectively. These did not recover within 20 min after exercise [20 Hz: -17.8 ± 16.5% (-27, -7%), *p* < 0.001, *g* = 0.826; 80 Hz: -14.0 ± 15.7% (-23, -3%), *p* < 0.001, *g* = 0.593; VFT: -17.9 ± 16.5% (-27, -7%), *p* < 0.001, *g* = 0.815]. While a significant time effect was detected for 20:80 Hz (*F*_(2.242,16)_ = 4.313, *p* = 0.018), follow-up tests revealed only a difference at POST-20 [-4.6 ± 7.1% (-1, -2%), *p* < 0.001, *g* = -0.043]. No significant time effect was observed for 20:VFT (*F*_(2.315,16)_ = 0.662, *p* = 0.543) (Table 2).

M-wave amplitude – there were significant time effects for SOL *M*_max_ (*F*_(1,16)_ = 0.121, *p* = 0.734) and MG *M*_max_ (*F*_(1.505,16)_ = 10.379, *p* = 0.002), where SOL *M*_max_ was reduced [-15.1 ± 14.8% (-40, 1%), *p* < 0.001, *g* = 0.458] at POST and did not recover within 20 min [-18.2 ± 18.6% (-41, 2%), *p* = 0.003, *g* = 0.433]. No changes in MG *M*_max_ were observed at POST [-3.8 ± 12.3% (-19, 5%), *p* = 0.211, *g* = 0.295], however, *M*_max_ was reduced at 10 min [-12 ± 12.1% (-25, -2%), *p* = 0.004, *g* = 0.560) and 20 min [-11.9 ± 11.8% (-25, -2%), *p* = 0.002, *g* = 0.578] after the exercise (Table 2).

## Discussion

Our primary aim was to describe the neuromuscular factors influencing the force loss and recovery following repeated bouts of high-intensity, yet submaximal, muscular efforts. The present data demonstrate that six sets of isometric plantar flexor contractions (3 s contraction/2 s rest) at 85% MVC with a 90-s rest between sets caused a significant loss of peak isometric torque (12.2 ± 6.5% in Experiment 1 and 16.9 ± 7.3% in Experiment 2), which occurred alongside changes at multiple sites along the neuromuscular pathway. Specifically, a significant loss of evoked tetanic torque was observed after exercise (Figure 5 and Table 2) which continued through the recovery phase (at least to our 20-min time point) and was likely a major factor limiting the number of contractions performed at the target level. Additionally, reductions in the M-wave-normalized EMG (EMG/M) immediately after exercise suggest a decrease in central/neural drive (Kalmar and Cafarelli, 2004b), which may have been underpinned by decreases in intrinsic cortico-motoneuronal excitability (decrease in MEP amplitude; Figure 3 and Table 1) and activation strength of PICs at the motor neuron (reduced self-sustained motor unit firing; Figure 5 and Table 2). Thus, the data indicate that a decrease in E–C coupling largely underpinned the loss of force after repeated, high-intensity muscular efforts, and that the loss may have been exacerbated immediately after the exercise by a loss of central/neural drive, possibly as a result of simultaneous decreases in corticospinal excitability and PIC amplitudes at the motor neurons.

In the present study, MEP/M measured in the relaxed muscle was unchanged by the exercise protocol, suggesting that there were no detectable changes in function of the cortico-motoneuronal pathway itself (Taylor et al., 2016) or motor neuron intrinsic properties (Heckman and Enoka, 2012) when assessed in the absence of facilitatory voluntary drive; it is also possible that different sites may have responded differently to the exercise, resulting in a lack of net change in MEP amplitude. Nonetheless, MEP/M measured during MVC was found to decrease following high-intensity exercise and then recover by 10 min. This time course was consistent with the time course of change in EMG/M, which also recovered by 10 min, indicating a possible association between the two variables. The temporal alignment provides some evidence that a change in corticospinal excitability measured during muscle contraction might be partly responsible for the loss in neural drive following high-intensity, submaximal exercise. This result is inconsistent with findings in plantar flexor muscles where MEP amplitude has been found to increase during submaximal sustained contractions (Hoffman et al., 2009) or remain unchanged when assessed during a MVC (Iguchi and Shields, 2012). Thus, it is possible that the type (sustained vs. intermittent) of contraction leading to the fatigue can profoundly affect changes in function of the corticospinal pathway. It may also, at least partly, reflect differences in the testing and analysis methods adopted such as the stimulus intensity used to evoke MEPs (Bachasson et al., 2016) and the sample size used for study. It should be noted that the single-pulse TMS only provides an indication of global changes within the whole cortico-motoneuronal pathway and thus it is unknown whether the decline resulted from changes in cortical or spinal sites, or a combination of the two.

The reduction in cSP found in the present study, considered to reflect a decrease in GABA_B_-mediated inhibition, has not been consistently shown in other studies, and although the change was small (i.e., -3.8 ± 10.2% immediately after exercise), the finding of statistical significance indicates that it was relatively consistent across participants and may thus be a true effect (i.e., not random error). Previously, cSP has been observed to either increase (Taylor et al., 1996; Yoon et al., 2011; Gruet et al., 2014; Goodall et al., 2017; Vernillo et al., 2017) or remain unchanged (Goodall et al., 2015; Jubeau et al., 2017) following fatiguing exercise. One possible explanation for the discrepancy is that, unlike during a sustained effort, the rest periods provided between individual repetitions as well as sets of exercise in the present study gave a chance for muscle reperfusion to occur and for a decrease in intramuscular pressure and metabolite concentrations (Anrep and Von Saalfeld, 1935; Hill, 1948; Cairns et al., 2017), which should reduce inhibitory group III/IV afferent activity and their effects on pain and pressure receptors (Amann et al., 2015). In turn, this reduction in inhibitory afferents might speculatively have allowed recovery (and indeed reduction) of the GABA_B_ inhibitory pathway and increased the neural drive to the motor neuron pool (Kidgell et al., 2017). It is of interest to determine whether these results are repeatable, and how the changes might influence the ultimate strength of the muscle contraction.

In addition to the loss in corticospinal excitability, motor neuron disfacilitation is also a possible mechanism contributing to the loss of neural drive (Taylor et al., 2000b; Hultborn et al., 2003); however, it has received little attention in the published literature. Alpha-motor neurons are strongly dependent upon facilitatory inputs to achieve a maximal discharge rate, and thus to produce high levels of muscular force (Hultborn et al., 2003). This facilitatory modulation occurs at the motor neuron dendrites (and soma) and is controlled by the interaction between descending monoaminergic drive and spinal circuits, especially including the Ia afferents (Heckman et al., 2005). It is well known that motor neurons rely on a PIC-mediated facilitatory system that increases synaptic gain in order to achieve maximal discharge rates and thus to produce maximal levels of muscular force (Hultborn et al., 2003; Heckman et al., 2005). To test whether a loss in PIC-mediated facilitation of the motor neuron occurs following fatigue, a combined vibration–electrical stimulation (VIB+STIM) protocol was used (Trajano et al., 2014). The VIB+STIM technique is believed to reflect PIC activation because the responses to it display many of the hallmarks of PIC-dependent change, such as muscle length dependence, where reflexive torque increases as the agonist muscle is lengthened (i.e., the antagonist is shortened), a wind-up effect during repeated muscle stimulations, a self-sustained torque phase after vibration cessation, and the inhibition of sustained firing by voluntary antagonist muscle activation (Trajano et al., 2014). Of course, the test might also theoretically be affected by other mechanisms, such as the spillover of serotonin (5-HT) from the somato-dendritic compartment of the motor neuron to inhibitory (5HT1A) receptors at the axon initial segment of the axon hillock, which would significantly reduce motor neuron excitability (Cotel et al., 2013; D’Amico et al., 2015). If this were the case, then MEP/M would also be expected to be reduced, particularly in the resting condition since the VIB+STIM technique was performed with participants in a relaxed state. Yet no changes were seen in MEP_rest_/M that might indicate a 5HT1A-dependent inhibition. It is also possible that vibration transmission through the muscle–tendon unit could be altered by fatigue. This might, for example, result in a loss of torque measured during vibration after each bout of stimulation; that is, the torque level would decrease but the proportion of wind-up should remain unchanged. However, wind-up was markedly reduced (Figure 6) after the exercise and recovered over time, suggesting that altered vibration transmission cannot completely explain the findings. Thus, the test appears to be robust against many physiological changes that can occur during fatigue, and was therefore considered to provide some indication of possible changes in PIC strength in the current study. Our results clearly show reductions in reflexive torque (*T*_vib_) production during vibration as well as reductions in the ability to sustain the torque without synaptic input (self-sustained torque; *T*_sust_) following the exercise (Figure 5). These results are considered to indicate an impairment in the ability to activate PICs or in their ultimate strength. *T*_vib_ and *T*_sust_ were statistically recovered by 10 min, suggesting that the effects of the fatiguing exercise were overcome relatively quickly. This temporal profile is also consistent with the change in EMG/M observed in both Experiments 1 and 2, indicating the possibility of an association between the changes in central/neural drive and the ability to activate PICs. More direct measurements are warranted (e.g., using the paired motor unit technique) in order to probe this possibility in greater detail.

Both MEP amplitude and PIC amplification were depressed at POST but recovered by 10 min. This aligns temporally with the loss and recovery of EMG/M and strongly suggests that the current fatiguing exercise protocol was sufficient to reduce neural drive during an MVC completed immediately after the exercise, but that these changes recovered within 10 min. These changes therefore cannot completely explain the prolonged loss of force that was observed at 20 min post-exercise, and other factors must be considered.

Reductions in tetanic torque were evident in 20 Hz, 80 Hz, and VFT tetanic stimulation conditions, suggesting that the muscle’s contractile capacity was compromised. The loss of force was similar across stimulation conditions and we were therefore unable to ascertain a specific process within the muscle that was predominant in the force decline. Previous studies have identified significant reductions in the 20:80 Hz ratio following fatiguing exercise such as downhill running (Martin et al., 2004) and concentric leg extension/flexion tasks (Hill et al., 2001) and this is believed to be caused by a reduced Ca^2+^ release or faster uptake of Ca^2+^ into the sarcoplasmic reticulum (MacIntosh and Rassier, 2002; Binder-Macleod and Kesar, 2005). However, the lack of relative differences in the torque change between the 20 and 80 Hz trains in Experiment 2 indicates a minimal effect of these mechanisms, although this may also be influenced by the position of the ankle (0°) and the type of muscle action (isometric) performed (Jones, 1996; Martin et al., 2004; Keeton and Binder-Macleod, 2006). The 20:VFT ratio is believed to represent changes in the muscle’s sensitivity to Ca^2+^, i.e., the ratio between the muscle’s myoplasmic Ca^2+^ concentration and the muscle’s force level (Abbate et al., 2002; Bentley and Lehman, 2005). The lack of change in 20:VFT suggests that a decreased Ca^2+^ sensitivity was not an overriding factor affecting force production following the fatiguing exercise. It is important to note, however, that because of the large compliance in the plantar flexor muscle–tendon unit (due particularly to the long Achilles tendon) the effect of the doublet stimulation on force production may be minimized when compared to other muscle groups such as the knee extensors (Binder-Macleod and Kesar, 2005; Mayfield et al., 2015) and this may have reduced our ability to detect small changes in the 20:VFT ratio. On the contrary, it might be the case that the doublet has a minimal effect in the plantar flexors and therefore a loss of the ability to use it, i.e., changes in Ca^2+^ sensitivity may not be important. Ultimately, a loss of force per cross-bridge (Edman and Lou, 1990; MacIntosh and Rassier, 2002) reduced force transmission from the contractile elements to the bone, or the cumulative effect of small (difficult to detect) changes in multiple force generating processes may have underpinned the force decline.

M-wave amplitudes were depressed following the fatiguing exercise protocol and did not recover within the 20-min recovery period in either Experiments 1 or 2, indicating that a change in fiber membrane excitability may have occurred and could have contributed to the prolonged loss of force following the exercise (Rodriguez-Falces and Place, 2016). However, evidence against a physiological effect of the M-wave amplitude reduction in the current study comes from the lack of change in the 20:80 Hz ratio. If the 20:80 Hz ratio is sufficiently sensitive to reflect changes in Ca^2+^ release from the Ca^2+^ release channels, then the lack of change in this parameter suggests that possible reductions in axonal hyperpolarization were insufficient to meaningfully impede muscle Ca^2+^ release and thus muscle function. Therefore, based on the present data, the potential changes in action potential propagation along the sarcolemma were not sufficient to meaningfully affect muscular function.

Caffeine ingestion was used to help identify potential physiological changes that might be responsible for the loss of force during the repeated, high-intensity muscular efforts, although it is commonly used (especially in athletic populations) to minimize the negative effects of fatigue and is worthy of study in its own right. Caffeine was found to produce a robust increase in muscle force production and neural drive (EMG/M) measured during MVC before and after the exercise. These results indicate that the greater torque attained under caffeine supplementation resulted from an increase in neural drive, which is consistent with evidence that caffeine exerts the majority of its effects on the nervous system (Snyder et al., 1981; Fredholm et al., 1999; Tallis et al., 2012). The mechanisms by which caffeine affects the nervous system are multifaceted, and include adenosine antagonistic behavior, which theoretically should increase MEP amplitude (Kalmar and Cafarelli, 2004a; Gandevia and Taylor, 2006), as well as the upregulation in noradrenergic and serotonergic drive, which could potentially increase PIC activation (Heckman et al., 2008) and thus improve excitability at the somato-dendritic end of the motor neuron (Cotel et al., 2013). However, corticospinal excitability (MEP) and motor neuron facilitation (sustained torque; *T*_sust_) were not statistically affected by the ingestion of caffeine; thus, it is likely that (1) caffeine had no discernible effect on motor neuron pre- or post-somatic sites and that caffeine exerted its effects through processes not tested within the present study or (2) that our tests were not sensitive enough to detect differences in corticospinal excitability and motor neuron facilitation between the non-caffeine and caffeine conditions. With specific reference to the effects of caffeine on PIC amplitudes, it is also possible that volitional (descending) neural drive is required in order for PIC activation to be sufficient; volitional descending drive is absent when using the VIB+STIM technique in the current study but is present when using the paired motor unit technique as in the study of Walton et al. (2002). Being that torque production was greater in Session 2 of both Experiments 1 and 2, it cannot be ruled out that learning effects may have influenced the MVC data. However, this was minimized through thorough familiarization of the plantar flexor test prior to study participation. In addition, as 8 of the 17 participants from Experiment 1 partook in Experiment 2, the chances of an order effect in Experiment 2 would have been reduced. It should be noted that the main purpose of the present study was to investigate physiological changes that might underpin the fatigue response (of which the caffeine condition was a part), and the effect of caffeine on force production itself was only a secondary outcome. Thus, placement of the caffeine condition after the non-caffeine session was considered necessary in the present study. It is also worth noting that the dosage of 3 mg kg^-1^ body mass used in the present study is lower than that used by other groups (Walton et al., 2002, 2003; Davis and Green, 2009; Behrens et al., 2015), where doses of 6 mg⋅kg^-1^ body mass were used. The lower dose was chosen to better represent the dosages that might commonly be ingested through the consumption of coffee, energy drinks, pre-exercise supplements, etc., in individuals performing strength training or in workers performing manual labor tasks. It is of interest to test a caffeine dosage greater than 3 mg kg^-1^ in future research, although its ecological validity might then be reduced.

## Conclusion

The present results indicate that the performance of repeated high-intensity, but submaximal, plantar flexor efforts results in a considerable and prolonged (>20 min) loss of force generating capacity which is likely to influence subsequent bouts of work. Significant decreases in evoked (tetanic) torque were observed at all time points, indicating that a decrease in the ability of the muscle to develop contractile force, irrespective of its activation level, largely explains the prolonged loss of voluntary torque. However, a specific process within the muscle was not identified; thus, no conclusions can be drawn as to the specific muscular mechanism/s responsible for the loss of torque. However, there was also a significant post-exercise loss of neural drive measured during MVCs, which were accompanied by decreases in corticospinal excitability (MEP amplitude) and motor neuron facilitation (self-sustained motor neuron firing). This loss of neural drive (and associated physiological changes) recovered quickly and would have only affected force production in the early period after the exercise. Caffeine ingestion improved muscle force production and neural drive (EMG/M) measured during MVC. However, its ingestion did not detectably affect corticospinal excitability or motor neuron facilitation pathways, so it is likely that either caffeine exerted its effects on neural mechanisms not tested within the present study or that our tests were insensitive to the small changes. Nonetheless, while fatigue resulting from high-intensity, intermittent muscular efforts is multifactorial, peripheral (muscular) changes can largely explain the loss of force and might therefore be targeted with exercise training, pharmaceutical, or other interventions in those who perform such tasks in the athletic or occupational roles.

## Data Availability

All datasets generated for this study are included in the manuscript and/or the supplementary files.

## Ethics Statement

The procedures performed during this research were approved by the Edith Cowan University Human Research Ethics Committee and were in agreement with the Declaration of Helsinki. Written informed consent was obtained from all participants.

## Author Contributions

All authors contributed to the conception and design of the study, manuscript revision, and read and approved the submitted version. BK, TP, and GR conducted the data collection. BK performed the data and statistical analysis, and wrote the first draft of the manuscript.

## Conflict of Interest Statement

The authors declare that the research was conducted in the absence of any commercial or financial relationships that could be construed as a potential conflict of interest.
